# Probe-based measurement of lateral single-electron transfer between individual molecules

**DOI:** 10.1038/ncomms9353

**Published:** 2015-09-21

**Authors:** Wolfram Steurer, Shadi Fatayer, Leo Gross, Gerhard Meyer

**Affiliations:** 1 IBM Research-Zurich, 8803 Rüschlikon, Switzerland

## Abstract

The field of molecular electronics aims at using single molecules as functional building blocks for electronics components, such as switches, rectifiers or transistors. A key challenge is to perform measurements with atomistic control over the alignment of the molecule and its contacting electrodes. Here we use atomic force microscopy to examine charge transfer between weakly coupled pentacene molecules on insulating films with single-electron sensitivity and control over the atomistic details. We show that, in addition to the imaging capability, the probe tip can be used to control the charge state of individual molecules and to detect charge transfers to/from the tip, as well as between individual molecules. Our approach represents a novel route for molecular charge transfer studies with a host of opportunities, especially in combination with single atom/molecule manipulation and nanopatterning techniques.

## Abstract (web summary)

Detection and manipulation of single charges in molecules are fundamental cornerstones in molecular electronics. Here, Steurer *et al*. demonstrate a technique with the use of an atomic force microscope that is able to resolve charge states and single electron charge transfer between molecules.

## Graphical abstract

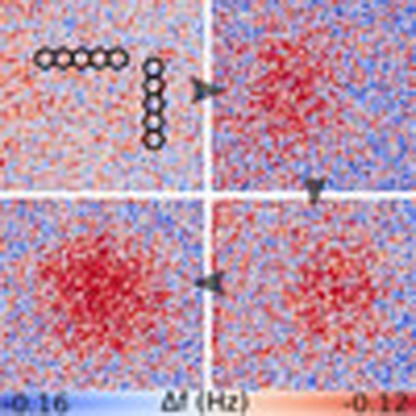

The functionality of molecules^1^,^2^,^3^,^4^ for use as electronics components^5^ is usually probed by conductance measurements, with the molecule sandwiched between metal electrodes^6^,^7^. Because the metal/single molecule/metal junction is sensitive to every microscopic detail, control over the atomistic configuration of the junction is essential for obtaining meaningful results. Using the tip of a scanning tunnelling microscope (STM), this degree of control could be achieved in a few cases^8^. In general, however, the atomistic details are unknown, and conductance histograms of thousands of measurements are statistically analysed to find the most probable configurations of the molecular junction. Today, a well-established scheme for measuring conductance as a function of gap separation and voltage is the break junction technique^9^,^10^,^11^, in which a molecular junction is repeatedly formed/broken, and a single molecule is located between the electrodes.

We circumvent the issue of dealing with stochastically defined junctions by investigating molecular charge transport in a planar geometry (Fig. 1). In our approach, single molecules and molecular assemblies are arranged on a bulk-like insulating film, and the tip of an atomic force microscope (AFM) is used to control and detect their charge state. Owing to the high sensitivity of the AFM, intermolecular charge transfer can be measured with single-electron sensitivity^12^,^13^,^14^, and charge state detection is also possible at large tip-molecule separations. While with our approach we demonstrate molecular charge transfer only in the weak-coupling regime, it does not preclude the investigation of molecular assemblies including also molecules more strongly coupled to electrodes. The combination of our method with atom/molecule manipulation^15^ and top-down nanopatterning techniques might open up the prospect of studying atomically defined molecular junctions in the future.

## Results

### Charge state control by AFM

Pentacene molecules adsorbed on 13 monolayer (ML) and as well as >20-ML thick NaCl films were selected as a model case, because well-ordered, defect-free NaCl films with large average terrace widths can be grown using standard epitaxy methods in ultra-high vacuum. At the larger film thicknesses (>20 ML )used, charge transfer to/from the substrate via the film is suppressed, and transport of single-electron charges is only possible between molecules as well as to/from the tip. At sufficiently large tip-molecule separations, charge exchange with the tip is also suppressed, and, hence, charges are conserved on the NaCl surface. Pentacene adsorbed on bilayer NaCl films has been intensively studied by STM^16^,^17^ and AFM^18^, and is readily identified via high-resolution AFM images obtained with pentacene-decorated tips^18^.

By positioning the AFM tip above a pentacene molecule and ramping the sample bias, we could reversibly switch the pentacene's charge state between negative, neutral and positive by attaching/detaching single electrons to/from the lowest unoccupied molecular orbital and highest occupied molecular orbital levels via the probe tip^19^, as schematically shown in Fig. 2a. Manipulation of the charge state entails a change of the local contact potential difference. Kelvin probe parabolas (dashed curves) corresponding to different charge states are shifted both horizontally and vertically. The direction of the horizontal shift of the parabola is a direct measure of the change of the local contact potential difference and enables discrimination of charge states^20^. The amount of the vertical and horizontal shift of the Kelvin probe parabolas depends also on the tip properties, that is, depending on the tip, we observe, for example, the vertical jump upon charging the lowest unoccupied molecular orbital to be also in the upward direction. In the experiments shown herein, charge state detection was also possible by the distinct frequency shifts at zero bias [Δ*f*(*V*=0 V)], which allowed us to acquire charge-distribution maps by recording constant-height Δ*f*-images at large tip-sample separations (*z*) with vanishing tunnelling probability across the vacuum gap. As an example Fig. 2g–j shows the sequential charging of four pentacenes spaced more than 50 Å apart including positive and negative charge states.

Figure 2b depicts an experimental Δ*f*(*V*) charging curve for the positive charging of a pentacene molecule by detachment of an electron from the highest occupied molecular orbital level. The electron's passage from the molecule to the tip appears as a jump-like feature in the Δ*f*(*V*) signal^19^. The reverse process, formation of the neutral pentacene, is shown in Fig. 2c. The jumps in Δ*f*(*V*) are observed at significantly different voltages in Fig. 2b,c which we attribute to the relaxation of the molecule-substrate system. We would like to point out that the observed voltage difference between the jump positions is not a direct measure of the relaxation energy, but varies strongly with the tip-pentacene distance and the bias ramp velocity. Moreover, the observed jump positions fluctuated statistically within a bias range of ±0.15 V, which depends also strongly on the tip-pentacene distance and the bias ramp velocity. Furthermore, for certain tip terminations, we detect a systematic shift of the jump voltages by more than 0.2 V, which we assign to different tip work functions^20^. Analogous charge state manipulation spectra for the formation and neutralization of a pentacene anion are shown in Fig. 2d,e, respectively.

### Coulomb repulsion between charged molecules

For assemblies of pentacene molecules, as shown in Fig. 3a–c, we could observe the effect of Coulomb interaction during charging. For a single pentacene molecule (Fig. 3d), the Δ*f*(*V*)-charging curve reveals detachment of an electron from the highest occupied molecular orbital orbital at *V*=−2.8 V (ref. 16). In the case of two adjacent pentacene molecules (8 Å centre to centre distance), as shown in Fig. 3e, the bias voltage for detaching the first electron remains the same, but the bias voltage has to be about 0.6 V more negative to detach a second electron. This value includes a voltage drop across the 13 ML NaCl film which we estimate to be at least 35% of the applied bias^21^,^22^. For comparison we calculated the corresponding Coulomb energy using a simple point charge model with charges located 2 Å above the NaCl surface and including the image charges in the NaCl to be 0.32 eV. The observation of Coulomb interaction can also reveal some useful information about the localization of charges within a molecular assembly. For example, for the three adjacent pentacene molecules shown in Fig. 3f, the measured Coulomb repulsion allowed us to infer the charge configuration. We attribute the first change in Δ*f* to charging of the middle or one of the outer pentacene molecules as the first step. From basic electrostatic considerations we deduce that the additional small decrease (compared with Fig. 3e) of the bias voltage necessary for detaching the second electron is only consistent with the two positive charges being on the outer pentacene molecules. This assignment is further confirmed by the larger voltage shift of 0.7–0.8 V for detaching the third electron. As this value is significantly smaller than the value of 1.2 V, that is, two times the charging value observed for the previous case of two pentacenes, it might be that screening by the neighbouring pentacenes has to be taken into account and is subject of further investigations. We would like to point out that for some of the transitions, within a small energy window, apparently repeated transfer of electrons between the tip and molecule is observed and we attribute this to a close tip-sample distance, that is, a small tunnelling barrier, in Fig. 3. This effect is not observed at larger distance. The small artifacts at higher voltages cannot be explained at the moment. The charging curves of the assembly of three pentacene molecules for three different tip positions is shown in Supplementary Fig. 2.

### Lateral charge transfer between individual molecules

By investigating a pair of pentacenes with a larger spacing, 18 Å centre to centre distance, and therefore a much lower intermolecular tunnelling rate compared with the case of Fig. 3 we can directly observe lateral charge transfer of single electrons (Fig. 4). These experiments were performed on a film with a thickness higher than 20 ML. While we could not experimentally determine the exact thickness of the film, we experimentally confirmed that charge transfer to the substrate was suppressed for more than 24 h.

**Figure 1:**
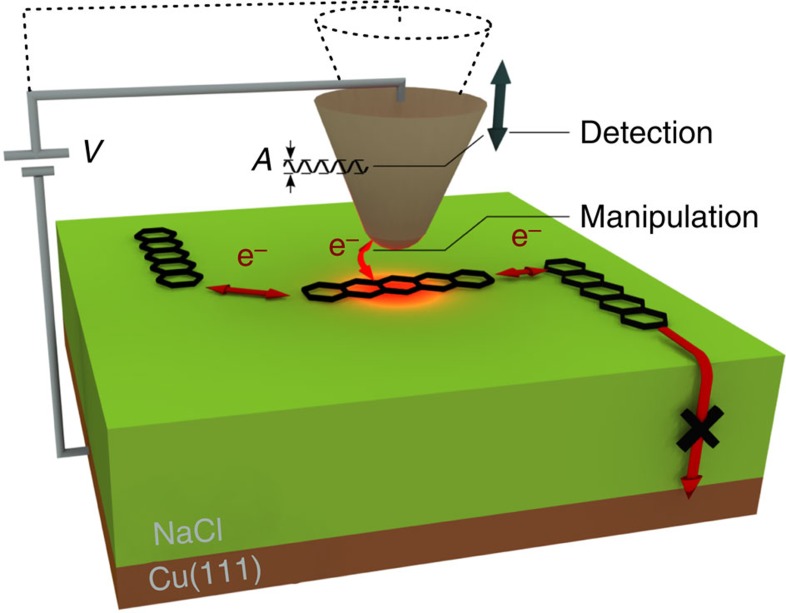
Experimental scheme. Schematic depiction of the experiment showing the definition of the sample voltage *V*, the oscillation amplitude *A* and transport of electrons from/to the tip as well as between adsorbates.

**Figure 2:**
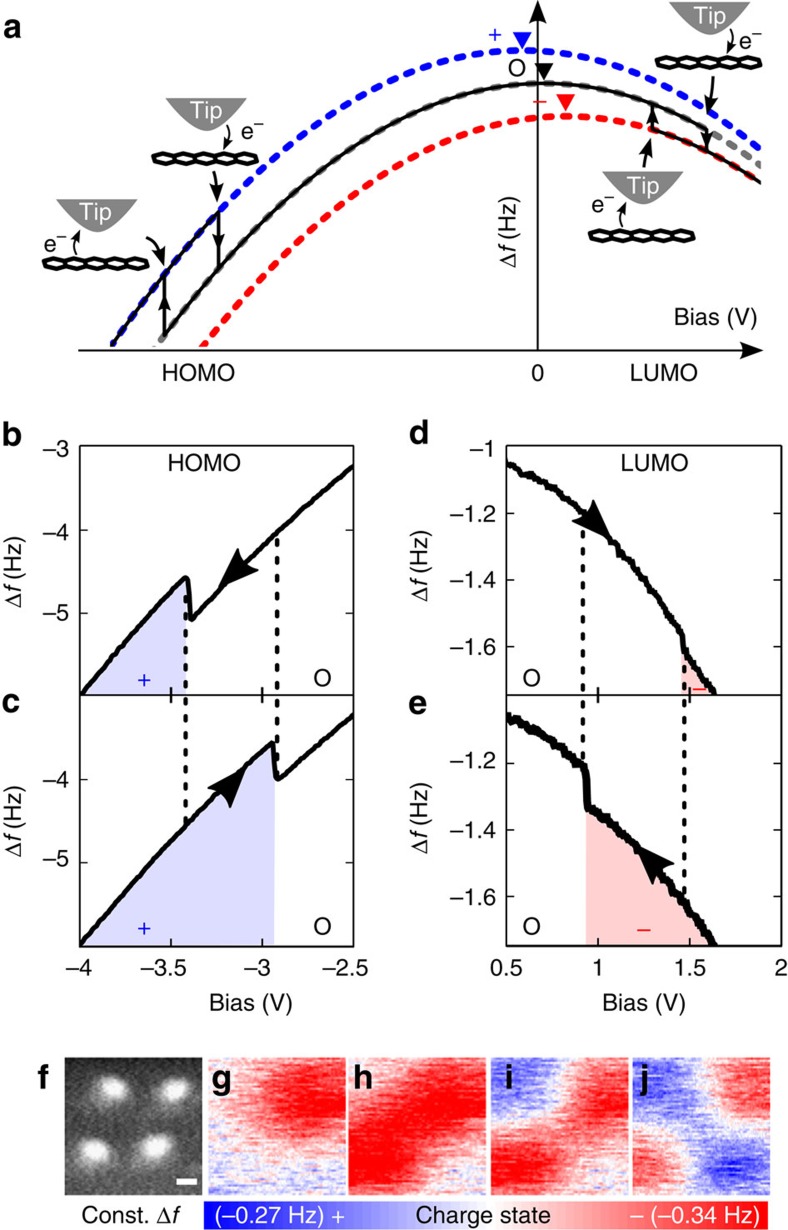
Charge state control by AFM. (**a**) Schematic depiction of a closed charge-switching cycle (neutral→negative→neutral→positive→neutral). The dashed parabolas visualize the change of the local contact potential difference (triangles). (**b**–**d**) Experimental manipulation spectra for detaching/attaching a single electron from the highest occupied molecular orbital (HOMO) (**b**) and to the lowest unoccupied molecular orbital (LUMO) level (**d**) and the reverse processes (**c**,**e**). The direction of the applied bias ramp is indicated by arrows in each case. (**f**) AFM topography image (Δ*f*=−2.9 Hz, *A*=0.8 Å, *V*=0 V) of four pentacene molecules. (**g**–**j**) Sequential manipulation of their charge state. After each manipulation step, the spatial charge distribution was mapped by recording Δ*f*-images at constant height (*z*≈18 Å, *A*=4.8 Å, *V*=0 V). (Higher resolved images are displayed in the Supplementary Fig. 1.) Scale bar, 20 Å.

**Figure 3:**
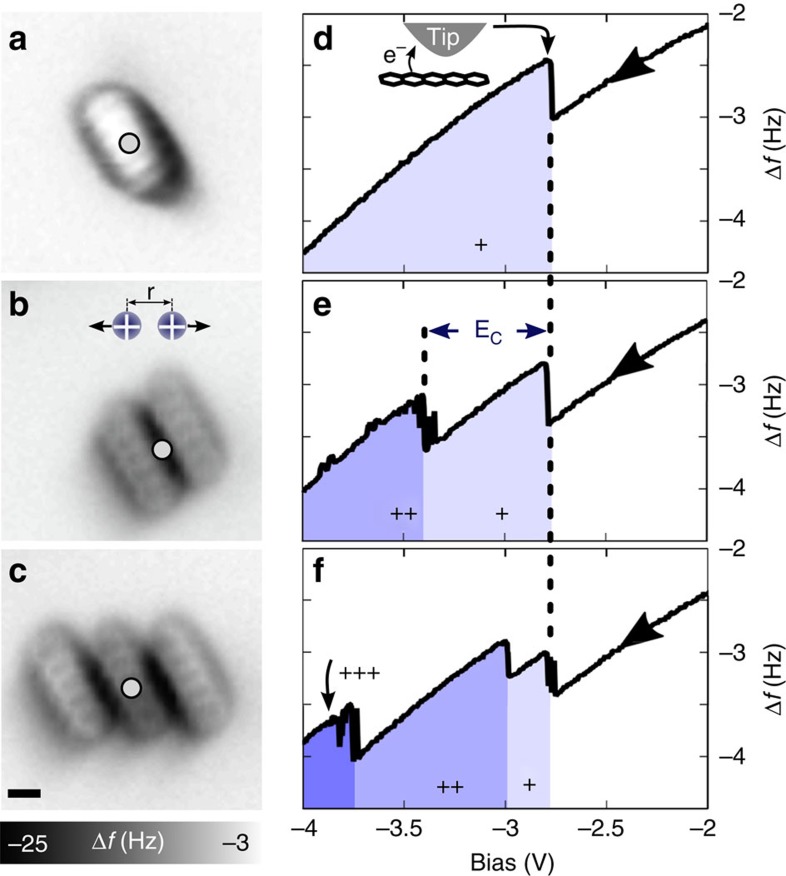
Coulomb repulsion between charged molecules. (**a**–**c**) Constant-height Δ*f*-images of different assemblies of pentacene molecules. (**d**–**f**) Experimental charging curves. The tip position is indicated by circles in **a**–**c**. Imaging details: *V*=0 V, *A*=0.8 Å, *z*=1.5 Å (**a**) and 3.6 Å (**b**,**c**); *z*=0 Å corresponds to an AFM set point (*V*=0 V, Δ*f*=−2.8 Hz) above NaCl. All images were obtained with a pentacene-terminated tip^18^. Scale bar, 5 Å.

**Figure 4:**
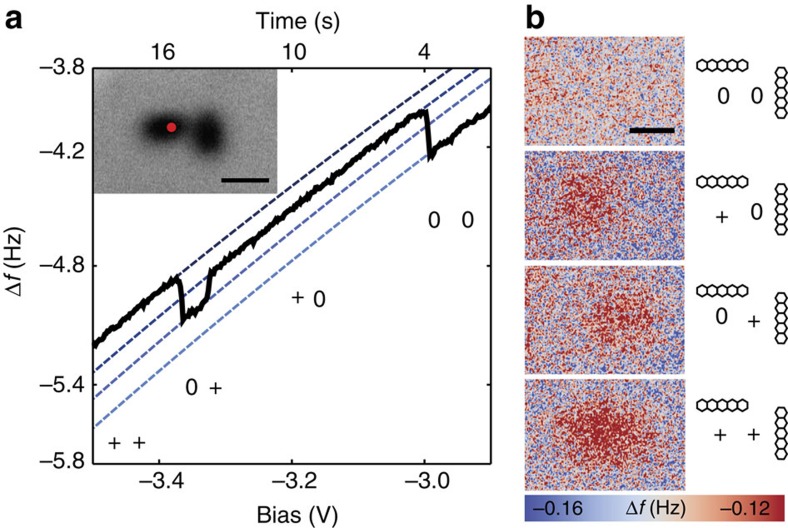
Lateral charge transfer between individual molecules. (**a**) The overview AFM image shown in the inset was taken in constant-height mode at a distance determined by a Δ*f* set point of 0.5 Hz at a sample bias of 0 V. The Δ*f*(*V*) curve was taken at a distance 6 Å further out from this set point. The time dependence is indicated on the top horizontal axis. The individual segments of the Δ*f*(*V*) curve have been fitted by four parabolas (dashed lines) corresponding to the four different charge configurations. (**b**) The constant-height AFM images map the four charge configurations and are taken at a distance 20 Å further out from the set point and at 0 V sample bias. A schematic representation of the corresponding charge configuration is shown next to each image. Scale bar, 20 Å.

The lateral charge transfer between the two pentacene molecules is exemplary shown by the Δ*f*(*V*) trace in Fig. 4a. Each of the four dotted Kelvin probe parabolas in this graph corresponds to a different charge configuration (00, +0, 0+ and ++) and can be assigned with a few simple considerations: (i) from the measurements on a single molecule we can deduce that the Kelvin probe parabolas shift upwards and further to the left upon each electron detachment (Fig. 2a) and (ii) the absolute value of the frequency shift sensed by the tip monotonically decreases with increasing distance between the charged object and the tip. By means of (i), the outermost Δf traces are directly identified as (00) and (++). Because of the position of the tip throughout the measurement, on top of the left molecule (see the inset of Fig. 4a), a change of the charge state of the left molecule (right molecule) in the pair will have the biggest (lowest) impact on Δ*f*. Thereby the Δ*f* traces corresponding to (+0) and (0+) are identified. To further confirm this assignment of the four charge configurations we have also directly imaged them using constant-height mode AFM, as shown in Fig. 4b. These images are obtained by ramping the bias voltage and performing the same Δ*f*(V) as in Fig. 4a, but manually stopping the ramp whenever a particular parabola has been reached. Such an experiment is possible because the average residence time in each parabola is at least on the order of a few seconds. Doing so enables us to perform constant-height Δ*f* maps, similar as in Fig. 2g–j. The imaging procedure consists of retracting the tip by 20 Å from the Δ*f* set point of 0.5 Hz at 0 V and imaging in the detection region of Fig. 1. Each image in Fig. 4b takes ∼20 min but provided that the tip is sharp enough yields enough resolution as to clearly determine where an electron has been detached from the pentacene pair.

With the charge states assigned, we can directly follow the lateral charge transfer by analysing the time dependence of Δ*f* shifts. First the pair is in the (00) state. At about −3 V the charge configuration switches to the (+0) state by detaching an electron from the left molecule to the tip. Further ramping the voltage to more negative values, we observe a shift to the (0+) state near −3.3 V. As we can rule out transfer of charge to the substrate, this evidences that the charge transfer from one molecule to the other has occurred. Finally at slightly more negative sample voltage another electron is detached from the left molecule and both molecules are charged now positively (++).

Apart from the simple two-molecule case described above, we have also studied an assembly of three molecules, resulting in a total of eight charge configurations, of which two are degenerate. Correspondingly six different Kelvin probe parabolas can be clearly identified, as shown in Supplementary Fig. 3, but as loss of charge to the substrate could unfortunately not always be ruled out, the interpretation of the data with regard to lateral charge transfer is not as well defined as in Fig. 4a.

## Discussion

We find that the tunnelling of charges to the substrate is suppressed on thicker films (Fig. 4). The fact that on these bulk-like NaCl films charges are stabilized is an essential prerequisite to study lateral charge transfer and more universal compared with energetic charge state stabilization of adsorbates using suitable substrate systems^23^,^24^. The observed transfer rate is determined by the tunnelling barrier between the molecules and the relaxation energy, which significantly shifts the energy levels of the orbitals and strongly reduces the transfer rate in this case^25^,^26^. We attribute the dominant part of the relaxation energy to the interaction with the ionic substrate. While we cannot provide an experimental value for the relaxation energy in the case of pentacene on NaCl, we would like to point out that on the same substrate but for Cl surface vacancies a relaxation energy close to 1 eV was calculated and a phonon mediated level broadening of 350 meV was measured^26^. This would result in a very strong decrease of the transfer rate due to the relaxation energy. Furthermore, due to the relaxation energy the transfer rate depends critically on the electric field of the tunnelling tip, as it shifts the energy of a molecular state directly below the tip more than the ones of neighbouring molecules. To demonstrate the importance of this local level shift we have also studied the transfer rate without influence of the tip. Under such conditions the charge transfer rate decreased by many orders of magnitude as compared to the situation in the presence of the field of the tip, as for example shown in (Fig. 4) (Tip-sample distance 12 Å, film thickness about 30 ML). This very small transfer rate for already closely spaced molecules made a reliable measurement of the distance dependence of the transfer rate without field not feasible in the present case.

In summary, we have shown that by placing the AFM tip in suitable positions above a molecular assembly, we are able to distinguish and control different charge configurations and follow the development of the charge states in time, identifying lateral charge transfer between the molecules, or between the molecules and substrate/tip. We expect that further experiments and more detailed modelling will allow us to get more quantitative information on the parameters controlling the lateral charge transport on an ionic substrate, in particular on the relaxation energy, the tunnelling barrier height and width, and the lateral distribution of the electrical field from the tip.

## Methods

### Sample preparation

Multilayer NaCl/Cu(111) films were prepared by evaporation of NaCl from a molecular beam evaporator onto a Cu(111) single crystal at room temperature. The NaCl/Cu(111) films were post annealed at 500 K for 5 min. Thirteen to twenty ML and thicker NaCl films were prepared. The Cu(111) single crystal was cleaned by repeated cycles of Ne^+^ sputtering and short annealing periods at 900 K before film preparation. Pentacene molecules were deposited at 5 K by vacuum sublimation from a direct current heated Si wafer. An overview image of the pentacene molecules on the 13 ML NaCl film is shown in Supplementary Fig. 4.

### Charge state manipulation by AFM

AFM experiments were performed at 5 K in a custom-built low-temperature STM/AFM based on the qPlus sensor design^27^, in which the vertical oscillation of a quartz cantilever is measured piezoelectrically (resonance frequency *f*_0_=30.1 kHz, spring constant *k*=1,800 Nm^−1^, quality factor *Q*=13,700). For charge state manipulation, the tip was first positioned at a distance ≈15 Å away from the surface. At this distance, the tunnelling probability through the gap vanishes, and charge states are inherently stabilized because tunnelling through the film is also suppressed^19^. Next, the tip was positioned above a pentacene molecule, and the tip-pentacene distance and the sample bias were varied following the sequence A→B→C→D for charging, or B→A→D→C for decharging, depending on the desired charge state manipulation. In step A, the tip was approached to the surface by 6 Å to increase the tunnelling probability across the gap. In step B, the sample bias was ramped from 0 V to a certain voltage (depending on the desired charge state). In step C, the tip was retracted by 6 Å from the surface to suppress charge transfer across the gap. In step D, the sample bias was ramped back to 0 V. All images have been taken at 0 V sample bias.

## Additional information

**How to cite this article:** Steurer, W. *et al*. Probe-based measurement of lateral single-electron transfer between individual molecules. *Nat. Commun.* 6:8353 doi: 10.1038/ncomms9353 (2015).

## Supplementary Material

Supplementary Information Supplementary Figures 1-4

## References

[b1] Liljeroth P., Repp J. & Meyer G. Current-induced hydrogen tautomerization and conductance switching of naphthalocyanine molecules. Science 317, 1203–1206 (2007). 17761878 10.1126/science.1144366

[b2] Quek S. Y. . Mechanically controlled binary conductance switching of a single-molecule junction. Nat. Nanotechnol. 4, 230–234 (2009). 19350032 10.1038/nnano.2009.10

[b3] Díez-Pérez I. . Rectification and stability of a single molecular diode with controlled orientation. Nat. Chem. 1, 635–641 (2009). 21378955 10.1038/nchem.392

[b4] Yee S. K. . Inverse rectification in donor-acceptor molecular heterojunctions. ACS Nano 5, 9256–9263 (2011). 22010940 10.1021/nn203520v

[b5] Joachim C., Gimzewski J. K. & Aviram A. Electronics using hybrid-molecular and mono-molecular devices. Nature 408, 541–548 (2000). 11117734 10.1038/35046000

[b6] Lörtscher E. Wiring molecules into circuits. Nat. Nanotechnol. 8, 381–384 (2013). 23736208 10.1038/nnano.2013.105

[b7] Ratner M. A brief history of molecular electronics. Nat. Nanotechnol. 8, 378–381 (2013). 23736207 10.1038/nnano.2013.110

[b8] Schull G., Frederiksen T., Arnau A., Sanchez-Portal D. & Berndt R. Atomic-scale engineering of electrodes for single-molecule contacts. Nat. Nanotechnol. 6, 23–27 (2011). 21076405 10.1038/nnano.2010.215

[b9] Reed M. A., Zhou C., Muller C. J., Burgin T. P. & Tour J. M. Conductance of a Molecular Junction. Science 278, 252–254 (1997).

[b10] Aradhya S. V. & Venkataraman L. Single-molecule junctions beyond electronic transport. Nat. Nanotechnol. 8, 399–410 (2013). 23736215 10.1038/nnano.2013.91

[b11] Sun L. . Single-molecule electronics: from chemical design to functional devices. Chem. Soc. Rev. 43, 7378–7411 (2014). 25099384 10.1039/c4cs00143e

[b12] Stomp R. . Detection of single-electron charging in an individual InAs quantum dot by noncontact atomic-force microscopy. Phys. Rev. Lett. 94, 056802 (2005). 15783674 10.1103/PhysRevLett.94.056802

[b13] Bussmann E. & Williams C. C. Single-electron tunneling force spectroscopy of an individual electronic state in a nonconducting surface. Appl. Phys. Lett. 88, 263108-1–263108-3 (2006). 10.1021/nl0620076 17090094

[b14] Brink M. *Imaging Single-Electron Charging In Nanostructures By Low-Temperature Scanning Force Microscopy* (PhD thesis, Univ. Cornell, 2007).

[b15] Custance O., Perez R. & Morita S. Atomic force microscopy as a tool for atom manipulation. Nat. Nanotechnol. 4, 803–810 (2009). 19966795 10.1038/nnano.2009.347

[b16] Repp J., Meyer G., Stojkovic S. M., Gourdon A. & Joachim C. Molecules on insulating films: scanning-tunneling microscopy imaging of individual molecular orbitals. Phys. Rev. Lett. 94, 026803 (2005). 15698209 10.1103/PhysRevLett.94.026803

[b17] Repp J., Meyer G., Paavilainen S., Olsson F. E. & Persson M. Imaging bond formation between a gold atom and pentacene on an insulating surface. Science 312, 1196–1199 (2006). 16728636 10.1126/science.1126073

[b18] Gross L., Mohn F., Moll N., Liljeroth P. & Meyer G. The chemical structure of a molecule resolved by atomic force microscopy. Science 325, 1110–1114 (2009). 19713523 10.1126/science.1176210

[b19] Steurer W. . Manipulation of the charge state of single Au atoms on insulating multilayer films. Phys. Rev. Lett. 114, 036801 (2015). 25659012 10.1103/PhysRevLett.114.036801

[b20] Gross L. . Investigating atomic contrast in atomic force microscopy and Kelvin probe force microscopy on ionic systems using functionalized tips. Phys. Rev. B 90, 155455 (2014).

[b21] Cockins L. . Energy levels of few-electron quantum dots imaged and characterized by atomic force microscopy. Proc. Natl Acad. Sci. USA 107, 9496–9501 (2010). 20457938 10.1073/pnas.0912716107 PMC2906850

[b22] Zheng N. . Electronic characterization of individual monolayer protected Au clusters by single electron tunneling force spectroscopy. Nanotechnology 21, 295708 (2010). 20601769 10.1088/0957-4484/21/29/295708

[b23] Repp J., Meyer G., Olsson F. E. & Persson M. Controlling the charge state of individual gold adatoms. Science 305, 493–495 (2004). 15273388 10.1126/science.1099557

[b24] Swart I., Sonnleitner T. & Repp J. Charge state control of molecules reveals modification of the tunneling barrier with intramolecular contrast. Nano. Lett. 11, 1580–1584 (2011). 21428431 10.1021/nl104452x

[b25] Marcus R. A. Electron transfer reactions in chemistry. Theory and experiment. Rev. Mod. Phys. 65, 599 (1993).

[b26] Repp J., Meyer G., Paavilainen S., Olsson F. E. & Persson M. Scanning tunneling spectroscopy of Cl vacancies in NaCl films: strong electron-phonon coupling in double-barrier tunneling junctions. Phys. Rev. Lett. 95, 225503 (2005). 16384232 10.1103/PhysRevLett.95.225503

[b27] Giessibl F. J. High-speed force sensor for force microscopy and profilometry utilizing a quartz tuning fork. Appl. Phys. Lett. 73, 3956–3958 (1998).

