# Supplementary material for: Probe-based measurement of lateral single-electron transfer between individual molecules
Source: Nat Commun. 2015 Sep 21;6:8353. doi: 10.1038/ncomms9353 (PMC4595756; doi:10.1038/ncomms9353)
Supplement: Supplementary Information — Supplementary Figures 1-4 [file ncomms9353-s1.pdf]

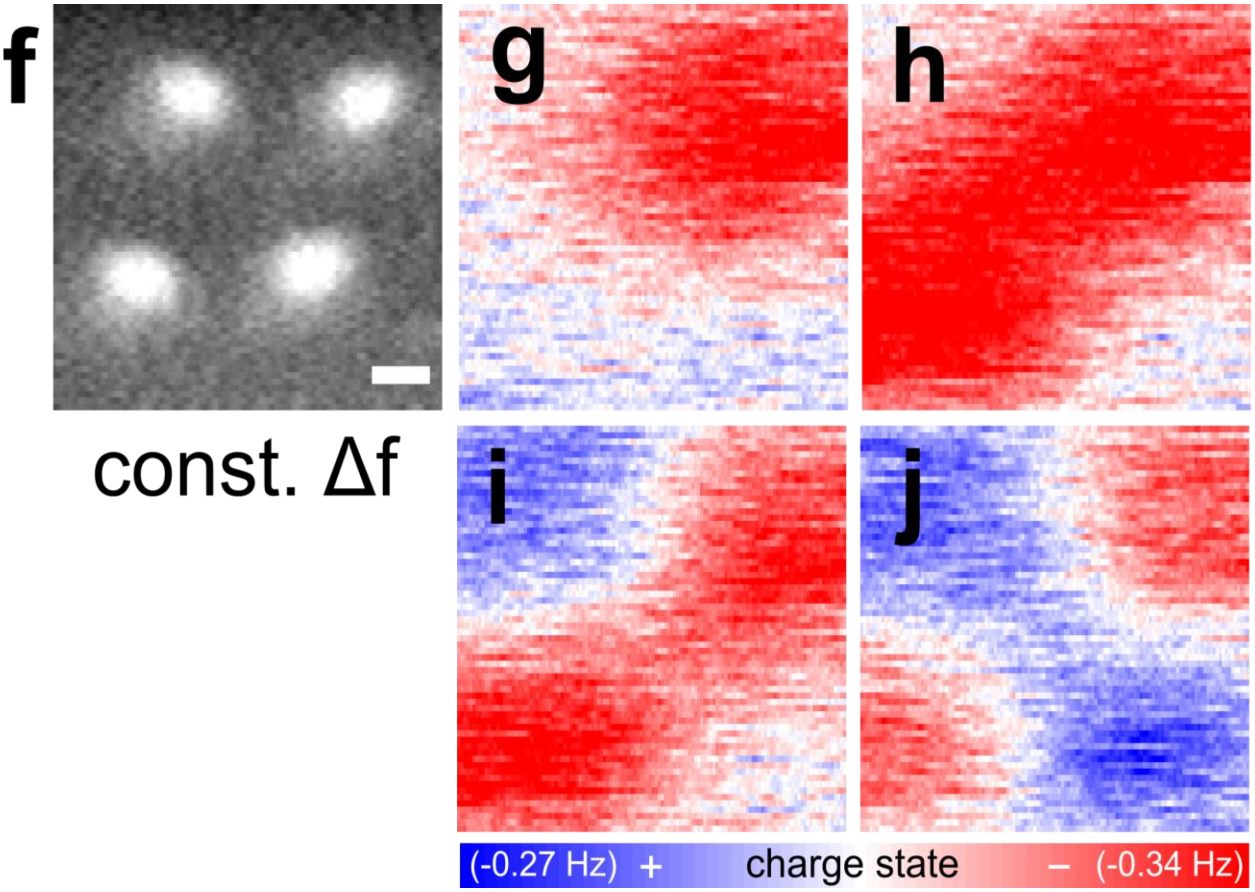

Supplementary Figure 1. Image of Fig. 2 of the main text shown with higher magnification.

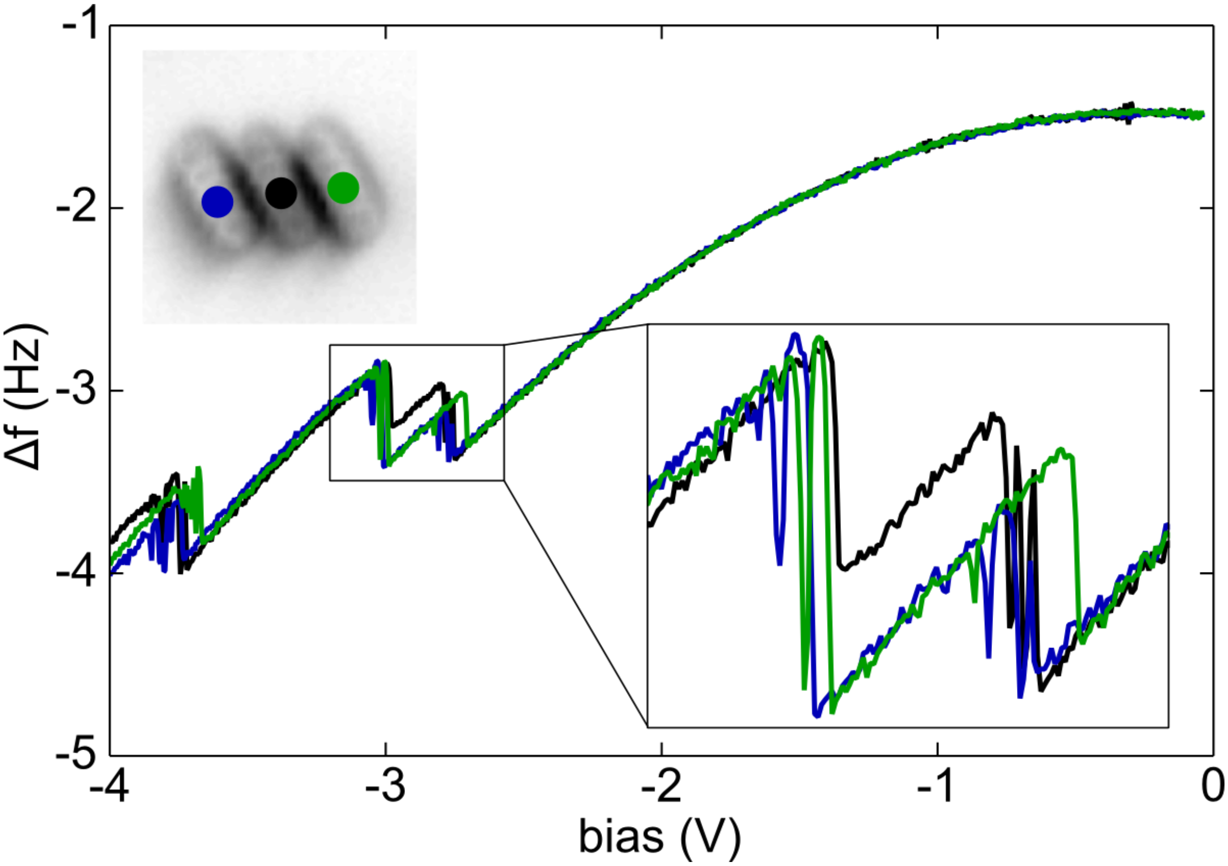

Supplementary Figure 2. Charging curves of an assembly of three adjacent pentacene molecules for different tip positions. Charging curves for the molecular assembly of Fig. 3c of the main text for three different tip positions, as indicated by the green, blue and black dots. A high-resolution AFM image is shown in the top left corner. Small changes in the detachment energies and jump heights are observed. The enlarged view in the inset shows a small Δf increase upon detachment of the first electron for the green and blue curve (tip positioned above the outer molecules), followed by a larger Δf increase after detachment of the second electron. The corresponding Δf increases for the black curve (tip positioned above the central molecule) are more similar.

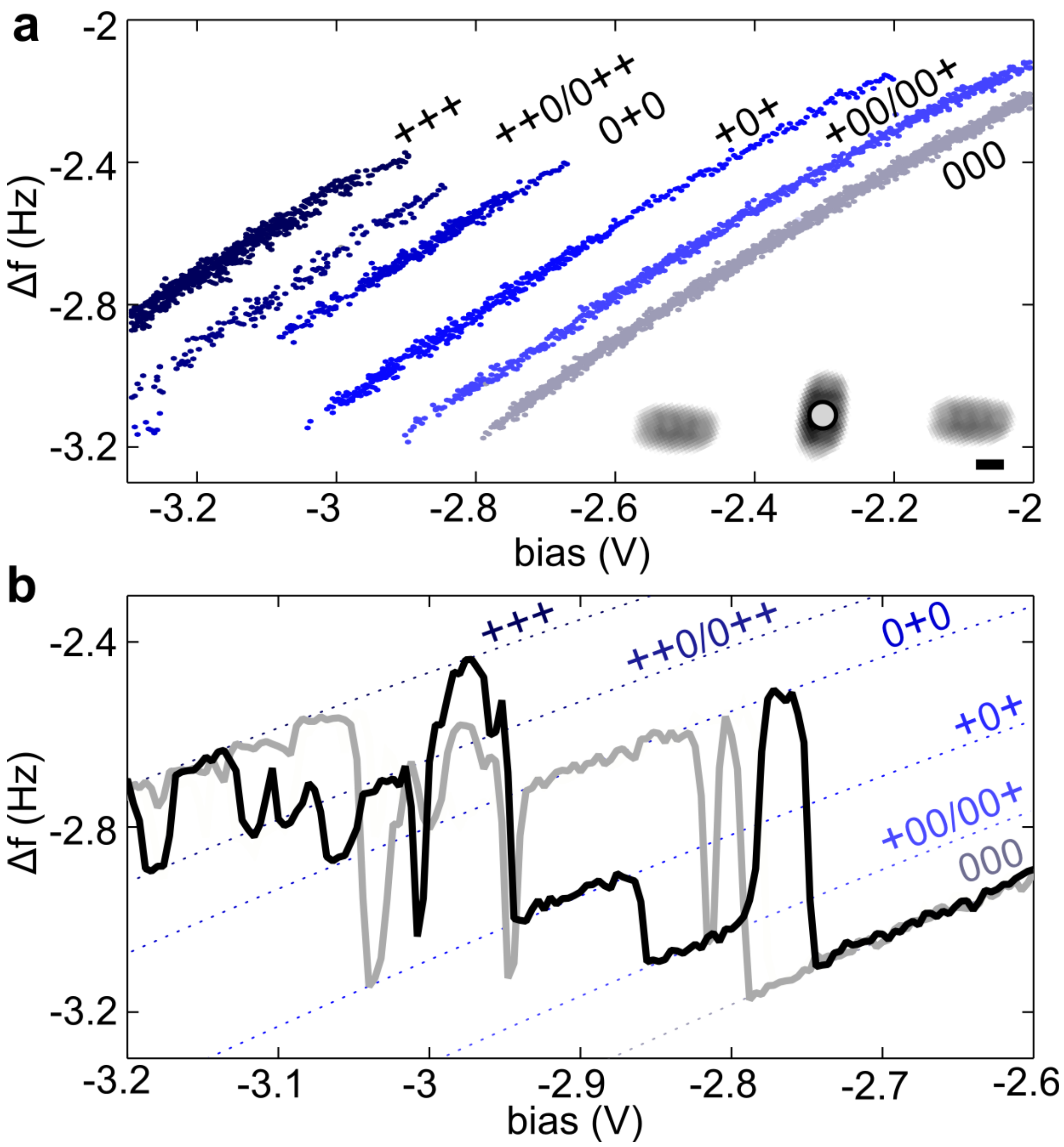

Supplementary Figure 3. Lateral charge transfer between individual molecules. This figure shows a molecular assembly of three pentacene molecules with larger intermolecular spacing and, therefore, lower intermolecular tunneling rates than the case evaluated in the main text. Data from six successively recorded charging curves (from 0V to -4V and back to 0V) recorded on the assembly of pentacene molecules depicted in the bottom right corner. Two exemplary charging curves are shown in (b). Distinct Δf(V) traces, corresponding to six distinguishable charge distributions, are labeled and colored differently. Data points corresponding to transitions between charge distributions are removed for the sake of clarity. The tip position is indicated by a black circle. The scale bar is 0.5 nm. The bias was ramped at a speed of -0.04 V s^{-1}. With the tip positioned above the middle pentacene molecule and due to

the symmetric arrangement of the outer pentacene molecules, whose centers are 2.6 nm away from the central molecule, six out of a total of eight possible charge distributions can be distinguished. The NaCl film thickness in this case is 20ML, but not as thick as in Fig. 4 of the main text, i.e. not fully ruling out possible charge transfer into the substrate. The data in (a) represents a superposition of a series of charging-curves such as shown in (b), which are falling onto 6 distinct $\Delta f(V)$ traces. For assemblies of pentacene molecules as the one shown in the lower left of (a), we can assign charge distributions to individual $\Delta f(V)$-traces through simple considerations: (i) Kelvin probe parabolas shift upwards and to the left upon electron detachment (a), (ii) the frequency shift sensed by the tip monotonically decreases with increasing distance and (iii) the detected frequency shift is, to a first approximation, additive for multiple charges of the same sign. By means of (i), the outermost $\Delta f(V)$ traces are directly identified as (000) and (+++). Because of the large separations between the molecules and the fact that the tip is positioned above the middle molecule, a change of the charge state of the middle molecule (one of the outer molecules) will have the biggest (least) impact on Δf. Therefore, the second-outermost $\Delta f(V)$ traces are identified as the (+00/00+) and (++0/0++) charge distributions. Finally, the (0+0) and (+0+) charge distributions can be assigned with the help of (iii): The $\Delta f(V)$ trace for (+0+) will be shifted relative to the (000) trace slightly more than twice as much as the (+00/00+) trace.

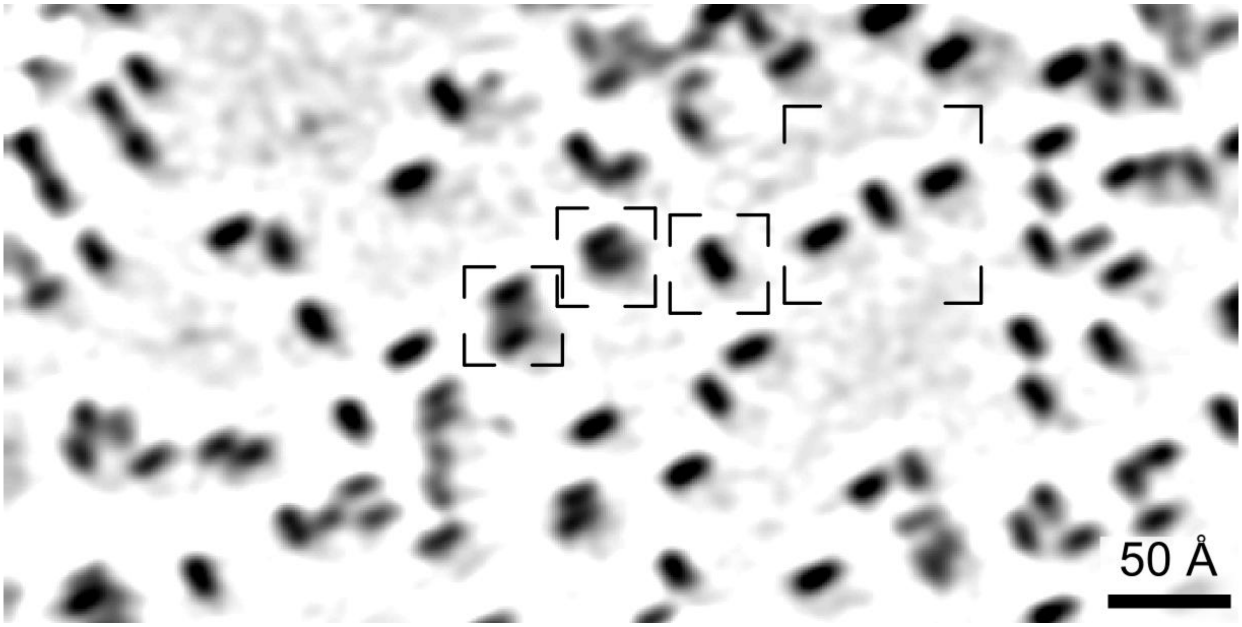

Supplementary Figure 4: Overview image. AFM overview image of pentacene molecules adsorbed on ~13 ML NaCl. The scan regions of the pentacene assemblies shown in the main text are marked (Figs. 3b and c of the main text are rotated by 90 degrees). Imaging details: const. Δf feedback, Δf = -2.8 Hz, sample bias of 0 V, tip decorated with a pentacene molecule. The image was Laplace filtered to enhance contrast.
